# Therapeutic analysis of Intrabeam‐based intraoperative radiation therapy in the treatment of unicentric breast cancer lesions utilizing a spherical target volume model

**DOI:** 10.1002/acm2.12140

**Published:** 2017-07-25

**Authors:** Madeline Schwid, Eric D. Donnelly, Hualin Zhang

**Affiliations:** ^1^ Department of Radiation Oncology Robert H. Lurie Comprehensive Cancer Center Northwestern University Feinberg School of Medicine Northwestern Memorial Hospital Chicago IL USA

**Keywords:** breast cancer, electronic brachytherapy, IB‐IORT, linear quadratic model

## Abstract

It is postulated that the outcomes in treating breast cancer with intraoperative radiotherapy (IORT) would be affected by the residual cancer cell distribution within the tumor bed. The three‐dimensional (3D) radiation doses of Intrabeam^TM^ (IB) IORT with a 4‐cm spherical applicator at the energy of 50 and 40 kV were calculated. The modified linear quadratic model (MLQ) was used to estimate the radiobiological responses of the cancer cells and interspersed normal tissues with various radiosensitivities. By comparing the average survival fraction of normal tissues in IB‐IORT and uniform dose treatment for the same level of cancer cell killing, the therapeutic ratios (TRs) were derived. The equivalent uniform dose (EUD) was found to increase with the prescription dose and decrease with the cancer cell infiltrating distance. For 50 kV beam at the 20 Gy prescription dose, the EUDs are 18.03, 16.49 and 13.56, 11. 29, and 9.28 Gy respectively, for 1.5, 3.0, 6.0, 9, and 15.0 mm of the cancer cell infiltrating distance into surrounding tissue. The dose rate of 50 kV is at least 1.87× higher than that of 40 kV beam. The EUDs of 50 kV beam are up to 15% higher than that of the 40 kV beam. The TR increases with the prescription dose, but decreases with the distance of cancer cell infiltration distance. Average TRs of 50 kV beam are up to 30% larger than that of 40 kV beam. In conclusion, IB‐IORT can provide a possible therapeutic advantage on sparing more normal tissue compared with the External Beam IORT (EB‐IORT) for shallowly populated unicentric breast lesion. Our data suggest that IB‐IORT dose size should be adjusted based on the individual patient's cancer cell infiltrating distance for delivering an effective dose, one dose‐fits‐all regimen may have undertreated some patients with large cancer infiltrating distance.

## 1 INTRODUCTION

The standard of care currently for locoregional treatment of breast cancer is breast conserving surgery followed by whole‐breast external beam radiotherapy (EBRT). It has been shown that postoperative radiotherapy significantly lowers local recurrence rates and translates into improved survival.^1^ Pathological analyses have shown that up to 90% of microscopic remainders of tumor cells after breast conserving surgery are observed in an area of 4 cm surrounding the macroscopic tumor edge, which is the region with the highest probability of local recurrence.^2^

An alternative to standard EBRT is intraoperative radiation therapy (IORT) in which at the time of breast conserving surgery a single dose of radiation is delivered.^3^ Unlike other types of radiotherapy, intraoperative radiotherapy (IORT) delivers a high single dose of radiation to the area around the tumor bed. Fowler^4^ postulated that breast cancer has an *α*/*β* ratio of 4, rather than the ratio of 10 that is characteristic of most squamous cell carcinomas. The lower *α*/*β* ratio corresponds to a lower radiosensitivity to low doses, favoring a high single‐dose treatment, such as IORT.^5^

The use of a 20 Gy dose of IB‐IORT (Carl Zeiss Surgical, Oberkochen, Germany) as a monotherapy following breast conserving surgery was compared to the standard of a 50 Gy dose of EBRT in the TARGIT‐A clinical trial.^6^, ^7^ In this still ongoing trial, patients were treated with either IB‐IORT or EBRT and outcomes were compared.^8^ Based on this trial and other studies, IB‐IORT has shown both benefits and drawbacks as compared to EBRT. Hypothesized benefits include the increased sparing of normal tissues and a shorter radiotherapy course from the 5 to 6 week course of EBRT. This shorter treatment option has been hypothesized to improve patient compliance, cost, and overall experience.^9^ Since the 20 Gy dose of IB‐IORT is biologically much greater than the fractional doses of EBRT, initially there was concern for an increase in toxicity, but studies have shown no treatment related mortality or excess morbidity including problems with wound healing, infection, or cosmetics.^3^, ^9^ One major difference between IB‐IORT and EBRT is breast changes such as fat necrosis and other distinct mammographic changes, which can complicate future diagnostic mammography, although usually, these changes are easily discernible from recurrent tumors.^9^ Because of this, IB‐IORT has been shown to be both safe and feasible.

The major drawback of IORT is a higher overall recurrence rate of breast cancer when compared to EBRT. With a median follow‐up of 29 months, Silverstein et al.^6^ reported that the 5‐year recurrence rates for the IB‐IORT versus EBRT patients in the TARGIT‐A trial were 3.3 and 1.3 %, respectively, *P* = 0.042. Local recurrence‐free survival was 92.9% for those treated with IB‐IORT and 92.5% for those treated with EBRT, *P* = 0.35. It is possible that with a select subgroup of patients, the difference in recurrence is small and acceptable and the benefits outweigh the risks.^6^, ^7^, ^10^ As this study will show, different treatment outcomes may have resulted from variation in cancer cell infiltrations.

The purpose of this study was to use a radiobiological model to evaluate the therapeutic impact of IB‐IORT on the cancer cells and normal tissue, and to compare IB‐IORT with the uniform dose EB‐IORT for the same spherical target volume without expanding the treatment margin. In this study, EB‐IORT is modeled as a hypothesized uniform dose radiotherapy which may be achieved by an external beam machine such as Mobetron (IntraOp Medical Corporation, 570 Del Rey Avenue, Sunnyvale, CA, USA). This study will not describe the particular dosimetric characteristics of Mobetron‐based EB‐IORT, but instead describe it as a single‐fraction uniform dose radiotherapy delivered immediately after lumpectomy. The radiobiological modeling results derived will be used to critically review the clinical data of TARGIT‐A clinical trial and to postulate how these factors may have related to the results found in the treatment and ongoing follow‐up. The conclusions derived from this study for breast cancer are considered to be applicable for other sites of IB‐IORT treatments.

## 2 METHODS AND MATERIALS

### 2.A Dosimetric and radiobiological effects of IB‐IORT

**Figure 1:**
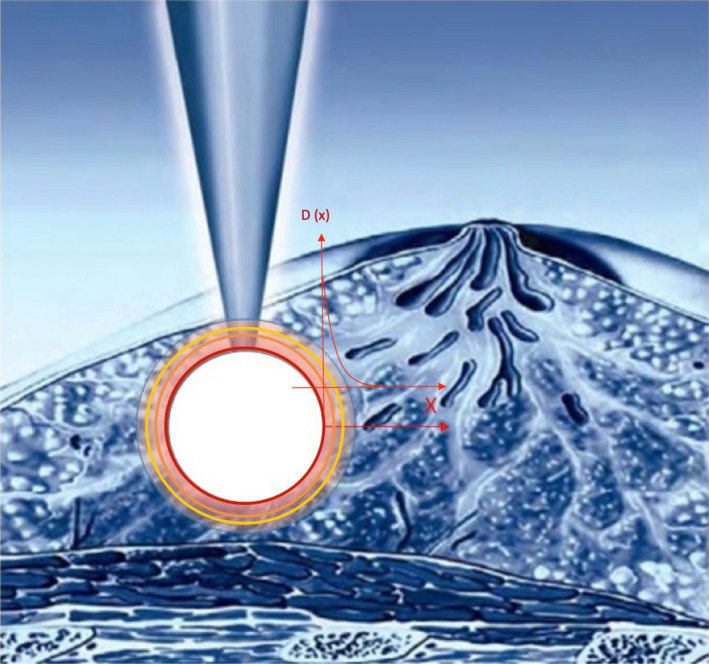
Schematic diagram of Intrabeam IORT for breast cancer. The target volume was assumed to be spherical. The dose drops with the distance exponentially. The cancer cell density variation with distance was assumed to drop and be a half‐Gaussian or linear.

**Figure 2:**
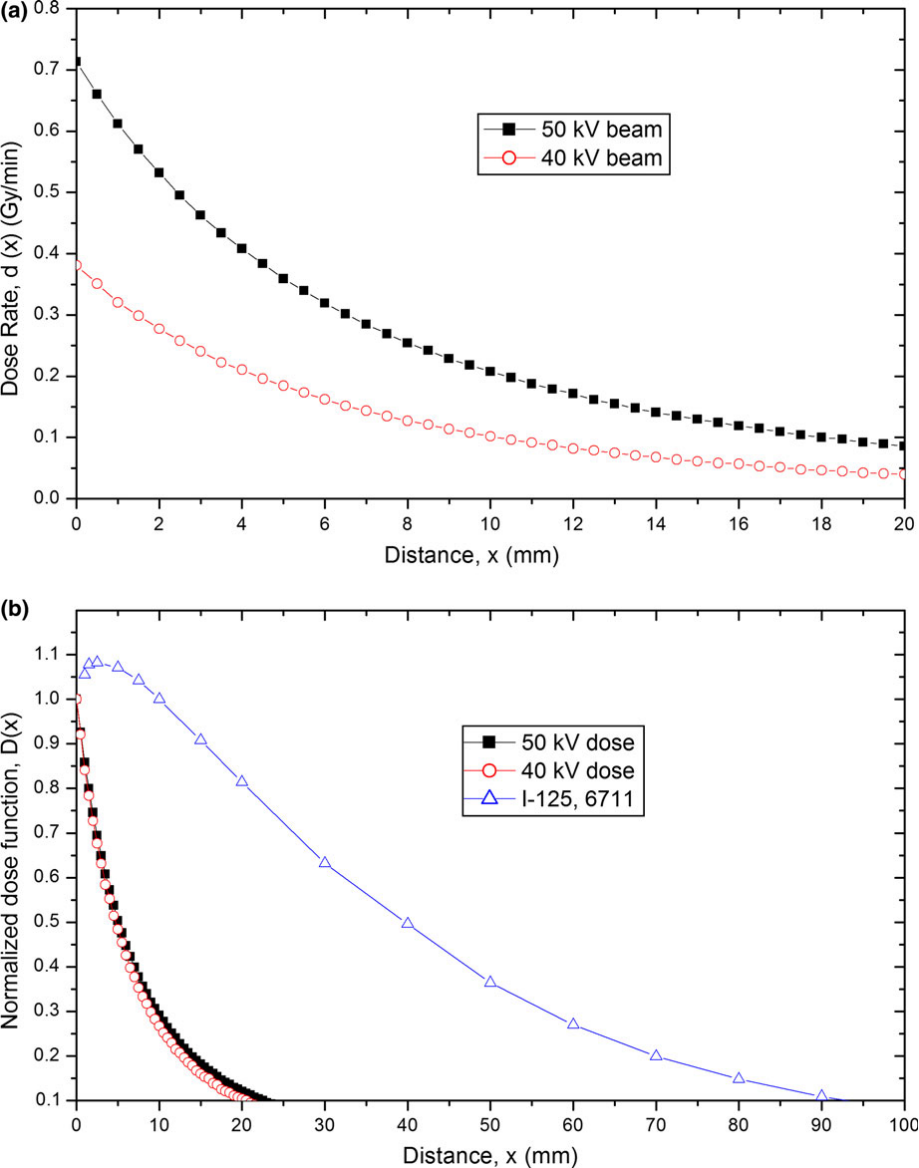
Radial dose rate function comparison between 40 and 50 kV beams of IB‐IORT (a); and normalized radial dose rate function comparison between IB‐IORT and ^125^I brachytherapy source (b). A 4‐cm spherical applicator was used in –IB‐IORT.

The IB‐IORT is designed to deliver a spherical dose to the surrounding tissue via its spherical applicator and electronic brachytherapy source (Fig. 1). Based on the commissioning data, the three‐dimensional (3D) radiation dose of the IB‐IORT using a 4‐cm diameter Intrabeam spherical applicator was calculated for the 50 and 40 kV beams. When using IB‐IORT, a large dose is usually prescribed at the applicator surface and the radiation dose curve drops very rapidly with distance. For example, for a 50 kV beam with a 4 cm spherical applicator, the dose drops to 7 Gy at 1 cm and to 2 Gy at 2 cm from the 20 Gy at the surface. Figure 2(a) shows the radial dose rate function comparison between 40 and 50 kV beams of IB‐IORT with a 4 cm spherical applicator. The dose rate of 50 kV beam is at least 187% of the 40 kV beam, this makes the 50 kV beam more efficient in the operation room treatment, since 50 kV beam will only use half of time of 40 kV beam to deliver the same dose. Figure 2(b) demonstrates the normalized radial dose function comparison between IB‐IORT (with a 4 cm spherical applicator) and a ^125^I brachytherapy source.^11^ The dose of IB‐IORT along the radial distance drops four times faster than that of ^125^I brachytherapy source, which indicated that IB‐IORT is suitable for dealing with superficially populated cancer. Figures 2(a) and 2(b) demonstrated that the dosimetric difference caused by the energy difference of two beams (40 vs 50 kV) is minor, but the dose rate difference will make a pronounced clinical impact. Because the dose drops to below 10% of the prescription dose at 2.5 cm depth, the doses to other adjacent normal structures such as the chest wall, heart, and lungs, are very low and not evaluated in this study.

The Intrabeam^TM^ machine can provide both the 40 and 50 kV beams, this study focuses the dosimetric metrics of IB‐IORT more on the 50 kV beam than on the 40 kV simply because the 50 kV is used in the TARGIT‐A trial.

For decades, the linear quadratic (LQ) model has been used for calculating tumor and normal cell survival fractions for a given dose of radiation therapy. If the cell repair and treatment time delivery is considered, this equation is expressed as^12^

(1) $$SF\left(x\right)\;=\;\exp(-\alpha ∙D\left(x\right)-\beta ∙G\left(\lambda ∙T\right)∙D{\left(x\right)}^{2})$$

Where *G(λT) = 2(λT + exp(‐λT) – 1)/(λT)* ^2^ is the dose protraction factor, *λ* is the repair rate, *T* is the delivery time, *SF(x)* is the survival fraction at depth x, *D(x)* is the radiation dose at depth x, and *α* and *β* are radiological parameters specific to the cell type. Recently, the accuracy of the LQ model for survival rates at doses greater than 12 Gy has come into question,^13^, ^14^ due to clinical observations have found that the classical LQ model overestimated cell killing at high doses. For this reason, a modified linear quadratic model (MLQ), introduced by Guerrero and Li^15^ for describing large dose radioresponses, is used in this study to estimate survival fractions of cell lines after the IB‐IORT. The MLQ model can be expressed as

(2) $$SF\left(x\right)\;=\;\exp(-\alpha ∙D\left(x\right)-\beta ∙G\left(\lambda ∙T+\delta ∙D\left(x\right)\right)∙D{\left(x\right)}^{2})$$

Where the dose protraction factor *δD(x)* was included. The parameter *δ* is a histological parameter that is calculated based on the effective repair rate, *λ*_*eff*_ *= λ + δD(x)*. The dose protraction factor describes the decrease in cell killing induced by treatment. As dose decreases, the MLQ model becomes the classical LQ model.

In our study, the survival fractions were calculated at the prescription doses of 8, 10, 15, 20, 25 Gy, respectively, for two types of breast cancer cells and three types of normal tissues. The 3D dose distributions were applied at the depth from 0 cm (at the spherical applicator surface) to the assumed cancer cell infiltrating distance, namely starting from the surgical excision — effectively the applicator surface and extending to certain cancer cell spreading distances. Average survival fraction of each cancer cell type at a given cancer cell distribution and prescription dose scenario was calculated based on the MLQ equation of survival fraction, cancer cell population, and volume (which was based on the concentric spherical shell), using the equation of

(3) $$\bar{SF}\;=\;\frac{\sum_{i-1}^{N}SF\left(x_{i}\right)∙V\left(x_{i}\right)∙f_{c}\left(x_{i}\right)}{\sum_{i-1}^{N}V\left(x_{i}\right)∙f_{c}\left(x_{i}\right)}$$

Where SF(x_i_) is the survival fraction at the depth x_i_ calculated by the MLQ model, V(x_i_) is the spherical shell volume for receiving dose D(x_i_), and f_c_(x_i_) is the cancer cell population at depth x_i_. The total cancer cell infiltrating distance was divided into N section with the step size of 0.5 mm. The cancer cell density variation with distance was estimated using either a half‐Gaussian or a linear distribution, which is discussed below. Normal cell population distributions were calculated for each scenario by subtracting the cancer cell population fraction from 1. It is noted that averaging cell survival in the eq. (3) is only applicable under the assumption of independent clonogenes and the cell communications were neglected. This assumption is more applicable to interspersed cancer cells than to normal tissues.^16^

Using the average survival fraction of the cancer cell, $\bar{SF}$, an equivalent uniform dose (EUD) for a given scenario was then calculated by solving the following equation:

(4) $$\bar{SF}=\exp\left(-\alpha *EUD-\beta ∙G\left(\lambda ∙T∙\delta ∙EUD\right)*{\left(EUD\right)}^{2}\right)$$

or

(5) $$\beta ∙G\left(\lambda ∙T+\delta ∙EUD\right)*{\left(EUD\right)}^{2}+\alpha *\left(EUD\right)+\ln\left(\bar{SF}\right)+0$$

The clinical meaning of EUD is, in order to achieve the same level of cancer cell killing across the target volume through a uniform dose radiotherapy, a uniform dose of EUD should be given. In addition, using EUD, 3D dose distribution of IB‐IORT, and normal cell population function f_n_(x_i_) (f_n_(x_i_) = 1‐f_c_(x_i_)), the average survival fractions of the three types of normal tissues (radiosensitive, moderate radiosensitive, and radioresistant) were, respectively, calculated in the EUD and IB‐IORT fields. The therapeutic ratio (TR) of this procedure was then defined^17^ and calculated by comparing the normal cell survival fractions between the IB‐IORT and EB‐IORT (namely EUD) with the equation of

(6) $$TR\;=\;\frac{{\bar{SF}}_{normal}\left(IB-IORT\right)}{{\bar{SF}}_{normal}\left(EB-IORT\right)}$$

A TR >1 implies that a greater number of normal cells survive in the IB‐IORT than in the EB‐IORT at the same rate of cancer cell killing, thus a therapeutic advantage of the IB‐IORT over the traditional EB‐IORT.

### 2.B Cell types and spatial distributions

The radiobiological response of breast cancer has been studied extensively in past decades.^4^, ^18^, ^19^ Based on the literature of modeling the radioresponse of melanoma and cervical cancer cells,^17^, ^20^ an acute responding and a slow responding breast cancer cell lines were chosen. The acute responding breast cancer cell line has an *α*/*β* ratio of 10, which is standard for squamous cell carcinomas. In a study by Guerrero and Li,^18^ a large number of clinical studies were pooled together to estimate the radiobiological parameters of breast cancer cells. They concluded that the *α*/*β* ratio of breast cancer cells might still be 10 Gy, with *α* = 0.3 Gy^−1^, and *β* = 0.03 Gy^−2^, and that the half‐life for repair of the cancer cells was 1 hour. The slow responding breast cancer cell line chosen in our study has an *α*/*β* ratio of 3.85, which was advocated by Fowler^4^ and agrees with derivations from other groups of authors.^4^, ^19^ For the normal tissues, a standard *α*/*β* ratio of 3.1 was chosen and *α* and *β* parameters were varied for the radiosensitive (SF(2 Gy) = 0.3), moderately radiosensitive (SF(2 Gy) = 0.5), and radioresistant (SF(2 Gy) = 0.7) tissue types. All the MLQ parameters for the five modeled cells are shown in Table 1. A value of 0.15 for *δ* and 0.693 for *λ* was chosen for all normal tissues and cancer cells.^15^, ^17^

**Table 1:** Breast cancer cells and normal tissue MLQ parameters

LQ parameters	Breast cancer cells		Normal tissue cells		
	Cell 1 (C1)	Cell 2 (C2)	Radiosensitive (N1)	Moderately radiosensitive (N2)	Radioresistant (N3)
α (Gy^−1^)	0.3	0.2	0.366	0.211	0.108
β (Gy^−2^)	0.03	0.052	0.118	0.068	0.035
δ	0.15	0.15	0.15	0.15	0.15
λ	0.693	0.693	0.693	0.693	0.693
α/β (Gy)	10	3.85	3.1	3.1	3.1
T (hr) (50 kV)	0.5	0.5	0.5	0.5	0.5
T (hr) (40 kV)	0.9	0.9	0.9	0.9	0.9

**Figure 3:**
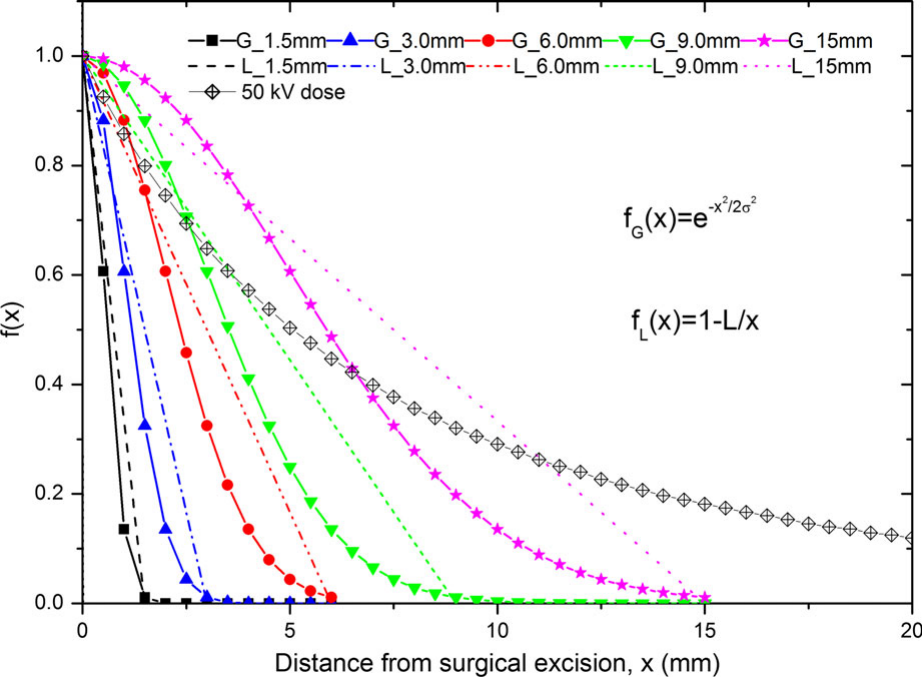
Assumed cancer cell infiltrations with half‐Gaussian and linear distributions at five different cancer cell spreading distances (1.5, 3.0, 6.0, 9.0, and 15 mm). The normalized IB‐IORT radial dose curve was also plotted to have a comparison.

Currently patient's residual cancer cell distribution in tumor bed after breast lumpectomy is unknown and a biopsy after lumpectomy may only tell the patient is cancer free at that specific biopsy spot. This is the reason an adjuvant therapy was always added after surgery. A study by Silverstein et al.^21^ had demonstrated that the likelihood of local recurrence in patients that were not treated with adjuvant radiotherapy decreased as tumor‐free surgical margin increased. Another study by Dunne et al. demonstrated that a 2 mm margin was superior to a margin less than 2 mm, but there was no significant difference seen with a greater than 5 mm margin.^22^ Other studies have made similar conclusions.^22^ These studies have also shown that ductal carcinoma in situ (DCIS) is almost always unicentric, the lesions seen in clinic are often large, and they present locally and lack stromal invasion and distant metastases. In the study by Faverly et al.^23^ showed that only 8% of DCIS have a multifocal distribution with gaps greater than 10 mm. In another study by Holland et al.,^24^ 81 of 82 mastectomy specimens of ductal carcinoma in situ showed only 1 region of tumor.^23^ Clinical observation indicates that most local recurrences occur at or near the primary lesion and likely results from inadequate surgical excision and residual disease, and all studies demonstrated that as the distance from the visible tumor border increases, the likelihood that microscopic residual cancer cells are present decreases. Considering the relationship between the recurrence possibility and the margin distance, two radially decreasing cancer cell distribution trends were assumed in this study (Fig. 3).

In the first trend, the cancer cell spatial distribution in the surgical tumor bed was assumed to be a half‐Gaussian distribution with a standard deviation of *σ* = 0.5, 1.0, 2.0, 3.0, or 5.0 mm, to imitate the cancer cell infiltration distances of 3 × *σ*, referred in the text as G_1.5, G_3.0, G_6.0, G_9.0, and G_15 mm, respectively. The half‐Gaussian cancer cell distribution cut off was set at 3 × *σ*, because at the distance of 3 × *σ*, the function percentage has dropped to less than 1.0% from 100% at the surface (0 mm). In this study, the minimum cancer cell density fraction was assumed to be as low as 0.01% at the excision surface (0 mm), so the density fraction at 3 *σ* would be 0.0001% (1.0% × 0.01%), which is almost undetectable.

In the second trend, the cancer cell density was assumed to linearly drop to 0 at the same distances from 100% at the excision surface. The linear distributions are referred in the text as L_1.5, L_3.0, L_6.0, L_9.0, and L_15 mm, respectively. Figure 3 shows the cancer cell distributions at five different half‐Gaussian and five different linear distributions. The maximum density fraction of cancer cells is at the depth 0 cm and was modeled in our calculations, respectively, for 10, 1, and 0.01% of total cells for each distribution, thus there are in a total of 30 different cancer cell population scenarios in this study.

It should be noted here that there is no experimental data to show that the actual residual cancer cell distribution after lumpectomy for most breast patients is half‐Gaussian or linear; we used these assumptions simply because the half‐Gaussian cancer cell distribution was found in other cancer sites.^25^ Also, a uniform cancer cell distribution seems unlikely given the clinical findings that the recurrence rate decreased with an increase in surgical margins and drastically declined at 2 mm margin.^21^, ^22^ In addition, a half‐Gaussian distribution is simple and it also follows the IB‐IORT dose fall‐off, therefore it is speculated that the cancer cell killing and normal tissue sparing would benefit from the IB‐IORT dose fall off with this pattern, since the region with the largest cancer cell burden will get the largest dose for intensive killing.

## 3 RESULTS

### 3.A Equivalent uniform dose

The Equivalent uniform dose (EUDs) for each cancer cell depth were calculated for a list of prescription doses ranging from 8 to 25 Gy for different sensitivities as shown in Table 2. The results demonstrate that the EUD is dependent upon the distribution of the cancer cell population and prescription doses, but not on the cancer cell radiosensitivities. In addition, since the 40 kV beam is also available, we provided a comparison between two energies in Table 2.

**Table 2:** EUDs of IB‐IORT for treating various distributions of breast cancer cells at different doses. Two types of breast cancer cells were evaluated

50 kV	EUD (Gy)									
Prescription DOSE (Gy)	G_1.5 mm		G_3.0 mm		G_6.0 mm		G_9.0 mm		G_15 mm	
	C1	C2	C1	C2	C1	C2	C1	C2	C1	C2
8	7.26	7.25	6.87	6.78	5.91	5.87	5.15	5.00	4.01	4.01
10	9.07	9.06	8.54	8.42	7.28	7.21	6.29	6.08	4.83	4.81
15	13.56	13.54	12.59	12.43	10.52	10.35	8.91	8.54	6.66	6.55
20	18.03	17.98	16.49	16.32	13.56	13.28	11.29	10.78	8.28	8.09
25	22.48	22.40	20.25	20.12	16.45	16.08	13.53	12.91	9.77	9.51

40 kV	EUD (Gy)									
Prescription DOSE (Gy)	G_1.5 mm		G_3.0 mm		G_6.0 mm		G_9.0 mm		G_15 mm	
	C1	C2	C1	C2	C1	C2	C1	C2	C1	C2
8	7.20	7.19	6.70	6.68	5.78	5.75	5.01	4.99	3.86	3.86
10	8.99	8.98	8.33	8.29	7.11	7.05	6.11	6.05	4.64	4.63
15	13.43	13.41	12.32	12.23	10.27	10.11	8.64	8.48	6.38	6.30
20	17.85	17.80	16.21	16.05	13.23	12.96	10.93	10.68	7.92	7.77
25	22.24	22.15	20.02	19.78	16.03	15.68	13.08	12.74	9.32	9.11

C1 is the acutely responding breast cancer (*α* = 0.3, *β* = 0.03), C2 is the slow responding breast cancer (*α* = 0.2, *β* = 0.052). G_3.0 mm means the cancer cell distribution was assumed to be half‐Gaussian, the cancer infiltrating distance is assumed to be 3 mm (3*σ =* 3 mm).

A further test indicated that when the cancer cell density fraction at the surface was varied (for example, from 10 to 0.01%) in any given cancer cell distribution, the EUD remains the same.

In addition, the efficiency of 40 kV beam was tested using the treatment time of 50 kV beam to calculate the EUD of 40 kV, thus the efficiency of two beams and the impact of dose rate can be evaluated. Using the G_3 mm lesion (the maximum infiltration is 3 mm in a half‐Gaussian distribution) and 4 cm spherical applicator as an example, EUDs of 50 kV beam for 10, 15, 20, and 25 Gy prescription are 8.54, 12.59, 16.49, and 20.25 Gy, respectively, but 40 kV would only reach the EUDs of 4.50, 6.71, 8.89, and 11.03 Gy, respectively, if the treatment times of 50 kV beam are used in the 40 kV beam treatments. Actually, under this 50 kV beam time condition, 40 kV would only deliver the prescription doses of 5.43, 8.01, 10.68, and 13.35 Gy, respectively, at the applicator surface.

### 3.B Therapeutic ratios of IB‐IORT

Table 3 shows the Therapeutic Ratios **(**TRs) of different breast cancer cells mixed in different normal tissues at the 6 mm cancer cell infiltration (standard deviation of *σ* = 2 mm). The TR of the IB‐IORT was found to be strongly dependent upon the prescription dose and cancer and normal cell radiosensitivities.

**Table 3:** Therapeutic ratios (TR) of IB‐IORT for two types of breast cancer cells. Each type of cancer cells were interspersed in one of three types of normal tissues. The assumed remaining cancer cell density is 0.01% at the surgical cavity surface; the cancer cell distribution is half‐Gaussian with a standard deviation of *σ* = 2 mm, namely G_6.0 mm. Two energies of beams (50 and 40 kV) were evaluated

	50 Kv								
DOSE (Gy)	C1				C2				Average
	C1N1	C1N2	C1N3	Average	C2N1	C2N2	C2N3	Average	
8	5.61	2.39	1.48	3.16	5.35	2.33	1.45	3.04	3.10
10	11.18	3.36	1.7	5.41	10.11	3.17	1.65	4.98	5.20
15	61.8	7.88	2.38	24.02	45.26	6.58	2.17	18.00	21.01
20	268.51	16.51	3.17	96.06	151.67	11.88	2.67	55.41	75.74
25	863.78	29.75	3.95	299.16	381.88	18.59	3.1	134.52	216.84

	40 kV								
DOSE (Gy)	C1				C2				Average
	C1N1	C1N2	C1N3	Average	C2N1	C2N2	C2N3	Average	
8	5.42	2.35	1.47	3.08	5.2	2.3	1.45	2.98	3.03
10	10.49	3.25	1.68	5.14	9.59	3.09	1.63	4.77	4.96
15	53.28	7.31	2.31	20.97	40.37	6.23	2.13	16.24	18.61
20	214.25	14.73	3.02	77.33	129.23	11.00	2.6	47.61	62.47
25	651.98	25.74	3.72	227.15	317.45	17.00	3.01	112.49	169.82

C1N1 represents the breast cancer cell 1 (acutely responding breast cancer, *α* = 0.3, *β* = 0.03) was interspersed in the normal tissue 1 (radiosensitive, *α* = 0.366, *β* = 0.118); C2N3 means that the breast cancer cell 2 (slow responding breast cancer, *α* = 0.2, *β* = 0.052) was interspersed in the normal tissue 3 (radioresistant, *α* = 0.108, *β* = 0.035); etc.

The TR was also found to be weakly dependent upon the cancer cell density within the target volume if the distribution function keeps the same. As the cancer cell density fraction at the surgical cavity surface was decreased from 10 to 0.01%, the TR was found to have changed only by 3%. This relationship is shown in table 4 which gives the average therapeutic ratios over the both types of cancer cells and three types of normal tissues at a cancer cell infiltrating distance of 3 mm in a half‐Gausssian cancer cell density distribution function (standard deviation of half‐Gaussian *σ* = 1 mm).

**Table 4:** Therapeutic ratios (TR) of IB‐IORT at 0.01, 1, and 10% of the remaining cancer cell density fraction at the surgical cavity surface for both the 50 and 40 kV beams. The standard deviation of the half‐Gaussian cancer cell distribution is *σ* = 1 mm, namely G_3.0 mm. The data are the average of both types of breast cancer cell lines (breast cancer cell 1 and 2) and three types of normal tissues (radiosensitive, moderate radiosensitive, and radioresistant)

	50 kV					
	Cancer cell 1			Cancer cell 2		
Dose (Gy)	0.01%	1%	10%	0.01%	1%	10%
8	2.16	2.17	2.20	2.19	2.19	2.22
10	3.16	3.17	3.23	3.18	3.18	3.24
15	10.12	10.15	10.39	9.55	9.57	9.80
20	35.25	35.34	36.27	29.18	29.25	30.02
25	112.55	112.80	115.91	76.61	76.81	78.90

	40 kV					
	Cancer cell 1		Cancer cell 2		
	0.01%	1%	10%	0.01%	1%	10%
8	2.21	2.21	2.24	2.19	2.19	2.22
10	3.21	3.21	3.27	3.13	3.14	3.19
15	9.68	9.70	9.94	8.94	8.96	9.17
20	30.64	30.71	31.52	25.76	25.82	26.50
25	86.99	87.22	89.57	64.92	65.08	66.84

In addition, as the cancer cell infiltrating distance was varied through varying the standard deviation of the half‐Gaussian distributions, the TRs of the IB‐IORT treatment were found to vary significantly. At the 15, 20, and 25 Gy prescription doses, IB‐IORT shows the largest TR in G_6.0 mm which translates into the largest normal tissue spring ratio in G_6.0 mm comparing with other cancer cell distributions (Table 5).

**Table 5:** Therapeutic ratios (TR) of IB‐IORT for a list of cancer cell distributions with the remaining cancer cell density fraction of 0.01% at the surgical cavity surface, in the 50 and 40 kV beams

50 kV					
Dose (Gy)	G_1.5 mm	G_3.0 mm	G_6.0 mm	G_9.0 mm	G_15 mm
8	1.51	2.16	3.10	3.26	2.76
10	1.83	3.16	5.20	5.34	3.99
15	3.36	10.12	21.01	19.18	9.97
20	6.85	35.25	75.74	59.83	22.92
25	14.43	112.55	216.84	152.59	47.14

40 kV					
Dose (Gy)	G_1.5 mm	G_3.0 mm	G_6.0 mm	G_9.0 mm	G_15 mm
8	1.59	2.21	3.03	3.15	2.63
10	1.95	3.21	4.96	5.04	3.70
15	3.63	9.68	18.61	16.64	8.56
20	7.38	30.64	62.47	48.01	18.19
25	15.23	86.99	169.82	114.68	34.87

G 3.0 mm means the cancer cell distribution was half‐Gaussian, the cancer cell infiltrating distance was 3 mm (3 = 3 mm).

### 3.C Comparison between the half‐Gaussian and linear cancer cell distributions

**Figure 4:**
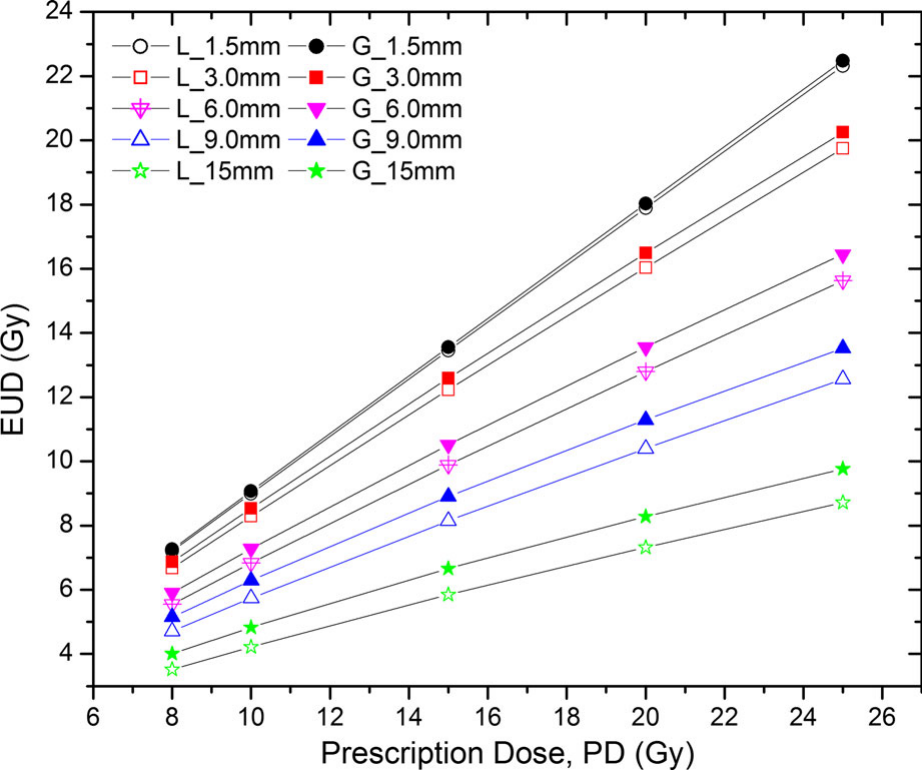
EUDs calculated by assuming half‐Gaussian and linear cancer cell distributions, respectively.

**Figure 5:**
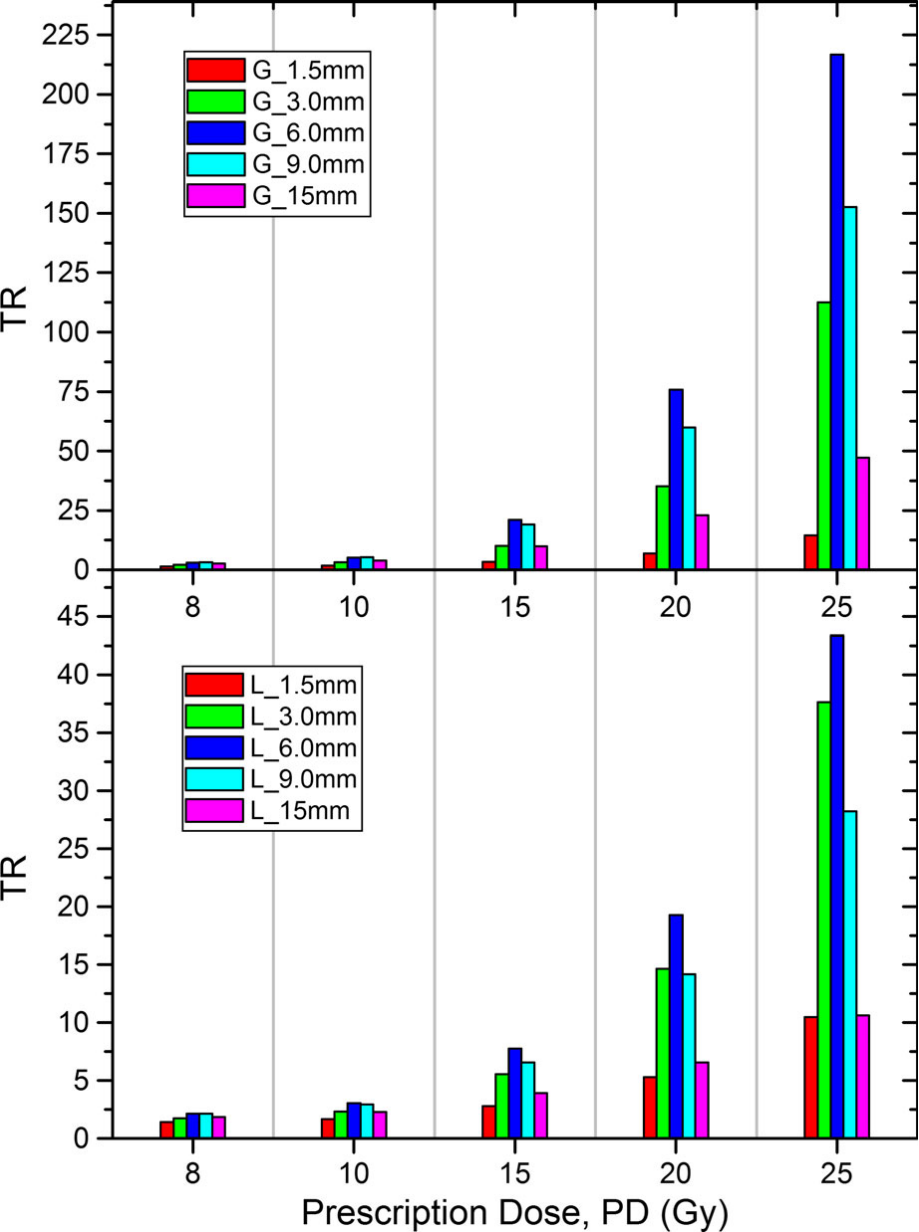
TRs calculated by assuming the half‐Gaussian and linear cancer cell distributions, respectively. Cancer cell density fraction at the excision surface was assumed to be 0.01%.

The EUDs and TRs calculated separately using the half‐Gaussian and linear cancer cell distributions are compared in Figs. 4 and 5 for the 50 kV beam. The half‐Gaussian distribution is seen to have a larger EUD and TR at the same cancer cell infiltrating distance and same prescription dose than the linear distribution. In addition, at the same prescription dose the 6‐mm cancer cell infiltrating distance (G_6 mm or L_6 mm) was found to have the largest TR among all cancer cell infiltrating distances. This phenomenon was seen in both the half‐Gaussian and linear distributions (Fig. 5).

## 4 DISCUSSION

The study evaluated the dosimetric metrics and normal tissue sparing effects of IB‐IORT, some important knowledge has been theoretically derived through radiobiology modeling. First remarkable finding is that, although the two types of cancer cells modeled have different radiosensitivities, our study indicated that the acutely responding cancer cell has almost the same EUD as the slow responding cancer cell, with a difference of only 0.5%. Second finding is the EUD is only weakly dependent on the cancer cell density fraction; the EUDs from the same cancer cell distribution were found to have varied only by 3% if the density fraction at the surface is decreased from 10 to 0.01%. Thus, the radiosensitivity and density fraction of cancer cells do not play an important role in the breast IB‐IORT treatments, if the EUD is considered to be the dosimetric end point. However, the EUD and TR were found to depend strongly on the cancer cell infiltrating distance, shallower breast cancer cells can be more effectively treated by IB‐IORT due to its unique dosimetric feature. Because the dose rate of the 40 kV beam is only about 53% of the dose rate of the 50 kV beam, the 50 kV beam is more efficient to treatment patients in the operation room.

Our study is not designed to investigate TARGIT A trial, but the results can be used to critically review the trial data. Despite many advantages such as convenience, better patient compliance and lower radiation dose to normal structures, the major drawback reported in the breast IB‐IORT is that, the TARGIT‐A clinical trial reported that the local recurrence rate was slightly higher in IB‐IORT than in EBRT, although this difference was not significant.^6^ The dosimetric and radiobiology reasons behind the high recurrence rate could be explained by our EUD data. As shown in Table 2 and Fig. 4, EUD is only a fraction (ranging from 9/10 at 1.5 mm of cancer infiltration to 4/10 at 15 mm of cancer infiltration) of prescription dose and the fraction decreases with the cancer cell infiltrating distance in the breast tumor bed. Because the EUD depends on the cancer cell infiltrating distance, a 20 Gy of the prescription dose such as that unanimously used in the TARGIT‐A trial may be enough for treating a tumor bed with 1.5 mm cancer cell infiltrating distance (G_1.5 mm or L_1.5 mm), but it is apparently not enough for treating a tumor bed with 15 mm cancer cell infiltrating distance (G_15 mm or L_15 mm). The EUD for the 15 mm infiltrating distance at 20 Gy prescription dose is only 8.28 Gy, comparing to 18.03 Gy for the 1.5 mm infiltrating distance at the same prescription dose. This means the IB‐IORT prescription dose may need to be significantly increased in order to achieve an effective EUD when the cancer cell infiltrating distance is large. From the EUD results it is understandable why the recurrence rate of IB‐IORT in TARGIT A trial is higher than the EBRT, because the one‐dose‐fits‐all TARGIT‐A trial regimen did not give a high enough dose in the cancer infiltrating regions of some patients.

Actually, the IB‐IORT dose can be adjusted for a suitable EUD. From Table 2, it is shown that a 20 Gy of prescription dose for treating a 3‐mm depth spherical lesion (G_3.0 mm) has the same EUD as the 25 Gy prescription for the 6‐mm depth spherical lesion (G_6.0 mm), the EUD of the both regimens is about 16.5 Gy. That means those two different pathological scenarios (3‐mm cancer cell infiltration vs 6‐mm cancer cell infiltration) could have a similar cancer cell control (EUD), if they are treated by two different dose regimens (20 Gy vs 25 Gy). In addition, it was found that in Table 5, in the 25 Gy regimen for G_6.0 mm a factor of 216.84× of more normal cells will be spared comparing with the uniform dose EB‐IORT, in the 20 Gy regimen (for treating G_3.0 mm) this factor is 35.25. Thus, it is very likely the patient's radiation toxicity would not increase remarkably at the 25 Gy dose since there is still a significant amount of normal tissue was preserved in IB‐IORT comparing with EBRT at the same level of cancer cell killing. Therefore, it is reasonable to speculate that, in TARGIT‐A clinical trial, a modest increase in the prescription dose (for example from 20 to 25 Gy) would likely increase overall tumor control probability and not risk adding significant radiation toxicities.

The results of using the linear cancer cell density distribution showed similar trends in EUD and TR, but since the IB‐IORT dose drops exponentially, the half‐Gaussian distribution showed a better conformality with the dose curve, and they were reflected by a comparable EUD and greater TR (Tables 2 and 5). This may imply that, if treated by the same prescription dose, a similar tumor control can be achieved for the both types of cancer cell density distributions (half‐Gaussian and linear) at the same cancer cell infiltrating distance, but the patients with the half‐Gaussian cancer cell density variation may have a lower complication rate, because a larger fraction of normal tissue was preserved in these patients. It should be noted that, although the advantage of cancer cell density conformality has been postulated in this study, the concept is still controversial if the dose needs to be adjusted for different cancer cell densities in order to spare more interspersed normal cells and thus reduce radiation toxicities.

Of the note, although the efficacy of EB‐IORT for breast cancer is limited by the irregular contour of deep cavity tumor beds as well as the close proximity of the critical structures, EB‐IORT with electron beam also has normal tissue sparing effect as seen in IB‐IORT, especially for low‐energy electron beams.^26^ This study only concerns the tumor bed less than 15‐mm depth, thus the normal tissue sparing effect and therapeutic advantage of EB‐IORT were not discussed as we know at this depth the electron beam EB‐IORT can be approximated as a uniform dose radiotherapy.

The results presented in this study are from the radiobiology modeling calculations, the uncertainties of data showing in the tables and figures are mainly from type B. Our experimental measurements indicate that the dosimetric uncertainty could be up to 10% for IB‐IORT source with spherical applicators, which is also consistent with the reported literature.^27^, ^28^ The major uncertainty sources of this study are: cancer cell distribution calculation at different depth (~3%), shell volume calculations at different depth (~3%), and the dose (~10%). Kirisits’ review report^29^ indicated that the uncertainty for the scatter dosimetric correction in the breast HDR balloon treatment is at least 7%. We estimate the uncertainty from the breast IB‐IORT scatter dosimetric correction is at the same level, also 7%. The cell culture study indicated the uncertainties of the cell radiosensitivity parameters of *α* and *β* could be up to 20%.^17^ We assume those components are not correlated, thus the total uncertainty of the EUD and TR is estimated to be 31%.

## 5 CONCLUSIONS

Based on the radiobiology modeling results presented in our data, the IB‐IORT has shown possible therapeutic advantages over traditional uniform dose EB‐IORT for postsurgical breast cancer treatment if the lesion is unicentric, the target volume is spherical and cancer cell density distribution is half‐Gaussian or linear. Because of more interspersed normal cells were spared, this may have helped mitigate the radiation toxicity.

Our results of the EUDs and TRs showed a significant variation among different cancer cell infiltrating distances and prescription doses; the breast cancer IB‐IORT can potentially improve the treatment outcome if the prescription dose could be adjusted for achieving a desired EUD based on the extent of cancer cell infiltrating distance within the tumor bed and surrounding tissue. Some institutions have started mapping the cancer cell density distributions for all tumor sites using either biopsy or functional imaging technologies. If the cancer cell distributions could eventually become known before patient's treatment regimen is designed, for example, if we know the maximum cancer cell infiltrating depth of breast cancer in breast conserving treatment, then the treatment plan can be tailored for the specific situation and prescription dose could be adjusted to ensure a sufficient killing of cancer cells, then the recurrence rate can be reduced and the toxicity level can be lowered, and finally the patient will benefit from the treatment. A personalized IB‐IORT treatment would likely improve treatment responses, reduce recurrence, and mitigate the radiation related complications.

## CONFLICTS OF INTEREST

The authors declare no conflict of interest.
